# Influence of Feeding Practices on Malnutrition in Haitian Infants and Young Children

**DOI:** 10.3390/nu10030382

**Published:** 2018-03-20

**Authors:** Belén Irarrázaval, Salesa Barja, Edson Bustos, Romel Doirsaint, Gloria Senethmm, María Paz Guzmán, Ricardo Uauy

**Affiliations:** 1 Division of Pediatrics, School of Medicine, Pontificia Universidad Católica de Chile, Santiago 8330023, Chile; belen.irarrazaval@gmail.com (B.I.); ruauy@med.puc.cl (R.U.); 2 Department of Pediatric Gastroenterology and Nutrition, Division of Pediatrics, School of Medicine, Pontificia Universidad Católica de Chile, Hospital Josefina Martínez, Santiago 8330023, Chile; 3 Department of Health Sciences (Nutrition and Dietetics), School of Medicine, Pontificia Universidad Católica de Chile, Hospital Josefina Martínez, Santiago 8330023, Chile; edsonbustos@gmail.com; 4 Klinik Saint Espri Health Center, Port Au Prince, HT 6311, Haiti; romeldorsaint@yahoo.fr (R.D.); gloriaasenethmm@yahoo.es (G.S.); 5 Fundación América Solidaria, Santiago 7500776, Chile; mariapazguzman@gmail.com

**Keywords:** breastfeeding, feeding practices, infant feeding, nutrition, malnutrition, pediatrics, primary health care

## Abstract

Infant malnutrition remains an important cause of death and disability, and Haiti has the highest prevalence in the Americas. Therefore, preventive strategies are needed. Our aims were (1) To assess the prevalence of malnutrition among young children seen at a health center in Haiti; (2) Examine adherence to infant feeding practices recommended by the World Health Organization (WHO) and the association to nutritional status. This cross-sectional study recruited children from the Saint Espri Health Center in Port Au Prince in 2014. We recorded feeding practices, socio-demographic data, and anthropometric measurements (WHO-2006). We evaluated 278 infants and children younger than two years old, aged 8.08 ± 6.5 months, 53.2% female. 18.35% were underweight (weight/age <−2 SD); 13.31% stunted (length/age <−2 SD), and 13.67% had moderate or severe wasting (weight/length <−2 SD). Malnutrition was associated with male gender, older age, lower maternal education level, and greater numbers of siblings (Chi^2^, *p* < 0.05). Adherence to recommended breastfeeding practices was 11.8–97.9%, and to complementary feeding practices was 9.7–90.3%. Adherence was associated with a lower prevalence of malnutrition. Conclusion: Prevalence of infant and young child malnutrition in this population is high. Adherence to WHO-recommended feeding practices was associated with a better nutritional status.

## 1. Introduction

The Ministry of Health and various international organizations performed several health surveys in Haiti between 2006 and 2012 [1,2,3]. In 2012, infant mortality in Haiti was 59–73 per 1000 live births, and under-five mortality was 88 per 1000 live births [4,5], the highest in the WHO region [4]. Although the prevalence of wasting has decreased to 4.1% (weight/height <−2 SD), great vulnerability and nutritional risk persists [6]. The prevalence of stunting (height/age <−2 SD) is 23.4% in children <6 years of age [1]. Infant feeding depends on cultures and customs, which can vary regionally and locally. The only study of infant feeding practices in Haiti [6] to date did not evaluate the association between such practices and the nutritional status of children.

Klinik Saint Espri Health Center is located in the Croix de Bouquetes commune, near Port Au Prince. This center opened in 2001 and is run by the American non-governmental organization (NGO) Haiti Medical Missions of Memphis [7]. Malnutrition is a frequent cause of consults and treatment program referrals at this center, but the magnitude of the issue is unknown. Low-cost evaluation methods using local staff and resources are needed to gather relevant data at the community level. This information could enhance planning, resource utilization, and intervention strategies. Studying and improving feeding practices is one important strategy [8].

The objectives of the present study were to assess the prevalence of malnutrition among infants and young children visiting the Klinik Saint Espri Health Center, to measure adherence to WHO-recommended feeding practices [9,10], and to evaluate the association between feeding practices and nutritional status.

## 2. Materials and Methods

We conducted a cross-sectional study at the Klinik Saint Espri Health Center in September 2014, using a convenience sample of infants and young children seen for acute morbidity, health check of newborns and infants, vaccination, or malnutrition within the Child Health Programs. We aimed to reach a sample of 200 children, based on a previous estimate of 323 visits per month. Recruitment was carried out by general and individual invitation in the waiting room. Children younger than two years old whose parents agreed to participate and who signed the informed consent form were included. Children who required immediate care due to severe disease or with clinical dehydration were excluded; excluded children were similar in age and sex to the final sample.

We developed a survey for the purposes of this study, translated into Haitian Creole by medical staff. The instrument was pilot-tested by two interviewers, and then final adaptations of the format and language were applied (Appendix A). The pilot instrument was administered to 20 children recruited from the waiting room, 2 months before the beginning of the definitive study. The survey evaluated five areas: (1) Identification and general characteristics of the patient (birth date was verified in the clinical records); (2) Brief social evaluation (maternal education, work, number of siblings); (3) 24-h dietary recall, collected once per participant by personal interview with each caregiver; to assess portion size, we used common plates and glasses obtained from local businesses and made models of common foods with painted plastic foam (Appendix B). For breastfed infants, it was not possible to estimate the volume of milk consumed, due to the variety of breastfeeding practices; (4) Use of nutritional supplements during the last week; and (5) Additional breastfeeding-related questions, including age at first breastfeeding, duration of exclusive breastfeeding, age at introduction to solid foods, and age at weaning from breastfeeding. The survey was conducted privately in an individual room by one of five trained interviewers; two were center staff and three were volunteers; all spoke fluent Haitian Creole. After the survey was completed, standardized anthropometry was performed, and the nutritional diagnosis was communicated to the child’s guardian. Children with wasting were immediately referred to the center’s malnutrition program. Daily reviews of the surveys were carried out to detect duplications and mistakes. Based on the information reported in the survey, specifically the 24-h dietary recall, indicators of feeding practices were calculated based on the methodology proposed by the WHO [6].

Anthropometry: Children were weighed without clothing, using a ADE non-digital infant scale, calibrated daily. A handmade wooden infantometer was used to measure supine length, with the head supported at one end, the torso and lower limbs extended, and feet flexed to 90° and supported by the lower-end stopper, to the nearest 0.1 cm. Head circumference (HC) was measured with a non-elastic measuring tape, fixed on the occiput and passing around the head and above the supraorbital ridges, as a marker of chronic undernutrition. Mid-brachial arm circumference was loosely measured at the mid-point between the acromion and the olecranon with the same non-elastic tape. Measurements were performed twice, rated, and repeated if inconsistencies were identified. Most of the measurements were performed by the first author (BI). The first author also trained the health center team (3 nurses and 1 paramedic) during a 4-h session and supervised in the application of the questionnaires (initially for the duration of the entire interview, and subsequently via intermittent daily observations). We excluded five subjects with incomplete data or inconsistent measurements who were not available for a new measurement.

The 2006 WHO Child Growth Standard was used; *z*-scores were calculated using the Anthro^®^ program [11,12]: for weight/age (zW/A), length/age (zL/A), weight/length (zW/L), head circumference/age (zHC/A), and mid-brachial circumference/age (zBC/A) [13]. The presence of edema was recorded. We used the 2006 WHO classifications for nutritional status [14]. A zBC/A value <−2 SD or measurement <125 mm was considered suggestive of malnutrition.

Adherence to feeding practice indicators: After applying the survey and before defining the nutritional status, the principal investigator (BI) calculated the indicator scores according to the WHO 2010 criteria for the child’s age [6].

Statistical analyses: Descriptive statistics of numerical variables were performed; distributions were verified using the Anderson-Darling test. Variables were expressed as mean (±SD) or median (range). Parametric (Student’s *t*-test) or non-parametric (Mann-Whitney) tests were used to compare results. Prevalence by sex and age were calculated (chi-squared test), and univariate correlation analyses were carried out to evaluate for associations. We evaluated the association between adherence to recommended feeding practices and nutritional status using the chi-squared test. The MINITAB-17^®^ program was used for statistical analyses. *p* < 0.05 was considered significant.

Ethics: The Research Ethics Committee of the Faculty of Medicine, Pontificia Universidad Católica de Chile; the medical directors of the Klinik Saint Espri Health Center; and the Haitian ambassador in Chile approved this study. All parents or guardians signed a written informed consent form, written in Creole. If the guardian was illiterate, a trusted person read the consent form (Ethics approval code 14-003).

## 3. Results

We assessed 278 infants, aged 8.08 ± 6.5 months (range: 13 days to 24 months); 41% were younger than six months, 31% were 6–12 months, and 28% were 13–25 months. Overall, 53.2% were female. There was no difference in age between girls and boys: 7.89 ± 6.05 and 8.31 ± 6.97 months, respectively (Mann–Whitney test, *p* = 0.59). Table 1 shows the characteristics and living conditions of the families. Most caregivers reported living near the Health Center, and the remaining came mainly from areas where the camps of the displaced populations following the 2010 earthquake are concentrated. Only 17.9% reported living in camps and/or housing made from lightweight materials. Parental employment was 34.5%, mostly small-scale trade jobs.

Median maternal age was 28 years range: (16–46 years). 43.5% of surveyed mothers had completed primary education, and 6.5% had no schooling. 15.35% of caregivers were unable to sign their names upon request (classified as illiterate). 35.7% of mothers reported having one child, 41.8% two or three children, and 22.5% four or more.

### 3.1. Prevalence of Malnutrition

Table 2 shows the prevalence of malnutrition according to various indices: 18.35% were underweight (weight/age <−2 SD); 13.31% had stunting (length/age <−2 SD); and 13.67% had moderate or severe wasting (weight/length <−2 SD). It is noteworthy that 10.8% had microcephaly and only 4.67% had low brachial perimeter.

**Figure 1:**
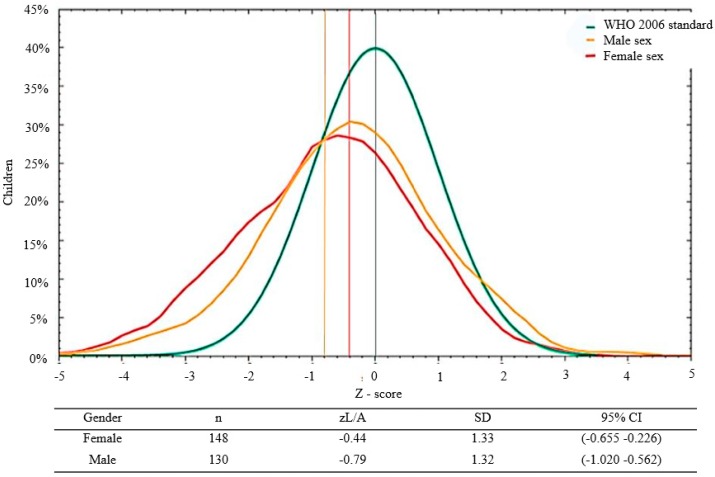
Distribution of L/A *z*-scores by sex* in 278 infants and young children seen at the Klinik Saint Espri Health Center, Port au Prince, Haiti (August 2014). Footnote: The green line indicates the WHO 2006 standard; the yellow line represents males and the red line females. * ANOVA, *p* = 0.029.

**Figure 2:**
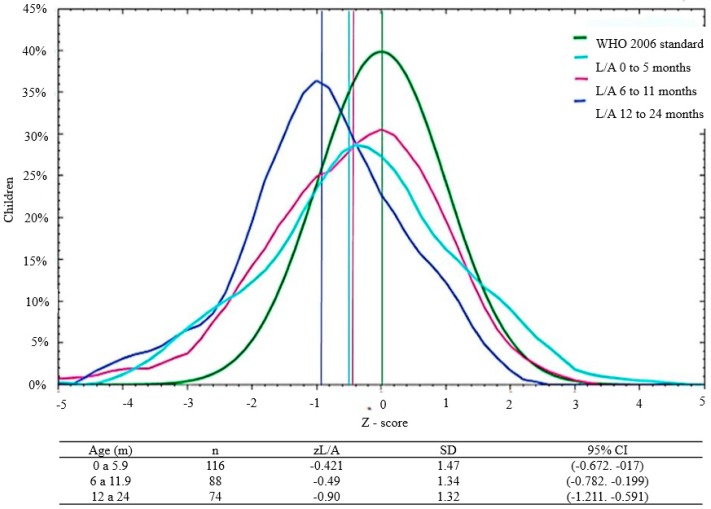
Distribution of L/A *z*-scores by gender* in 278 infants and young children who attended the Klinik Saint Espri Health Center, Port au Prince, Haiti (August 2014). Footnote: The green line indicates the WHO 2006 standard; the light blue line represents children between 0 and 6 months, the lilac children between 6 and 11 months, and the blue children between 12 and 24 months (* ANOVA, *p* = 0.038).

The curves for the anthropometric indices were displaced to the left relative to the WHO standard, both globally and by age and sex (Figure 1 and Figure 2). Importantly, only 30.6% of the infants with zW/L <−2 SD (acute malnutrition or wasting) were enrolled in the Malnutrition Program of the Health Center. None of the children had edema.

### 3.2. Malnutrition by Sex and Age

We found a non-significant trend of lower zW/A in male versus female infants, at −1.01 ± 1.44 versus −0.716 ± 1.29, respectively (Student’s *t*-test, *p* = 0.07). We also observed a non-significant trend of lower *z* W/A in older infants: 0–5 months: −0.69 ± 1.46, 6–11 months: −0.85 ± 1.32, and 12–24 months: −1.1 ± 1.25, respectively (ANOVA, *p* = 0.13). Male infants had a significative lower *z* L/A compared to females (Figure 1), and older infants had a lower *z* L/A than younger infants (Figure 2). There was a non-significant trend for lower *z* W/L in male versus female infants: −0.61 ± 1.15 versus −0.73 ± 1.38, respectively (Student’s *t*-test, *p* = 0.42). Finally, there was a tendency towards higher *z* W/L in younger infants: −0.55 ± 1.39 (0–5 months), −0.70 ± 1.14 (6–11 months), and −1.80 ± 1.20 (12–24 months) (ANOVA, *p* = 0.38).

### 3.3. Malnutrition According to Family Characteristics

**Figure 3:**
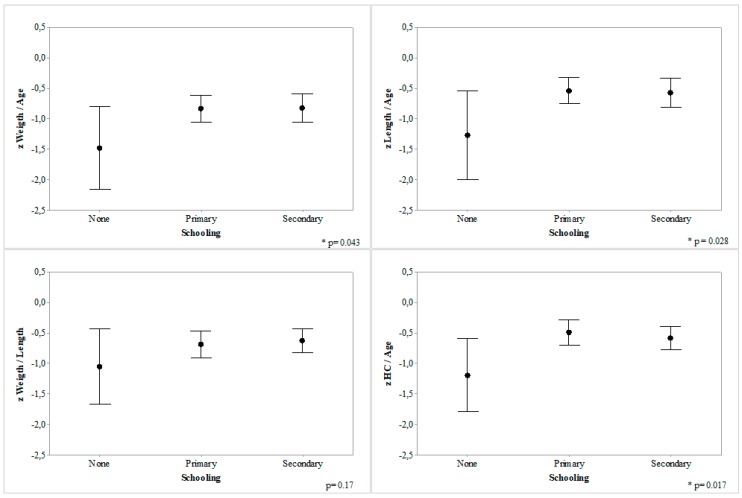
*z*-score of anthropometric indices according to maternal education, in 278 infants seen at the Klinik Saint Espri Health Center, Port au Prince, Haiti (August 2014). Footnote: 95% Confidence Interval, medians represented by black circles. * *p* < 0.05, ANOVA. Comparing children from mothers lacking formal education with those with mothers having primary or secondary education.

Children of mothers lacking formal education had significantly lower *z* W/A, *z* L/A, *z* HC/A, and *z* BC/A scores than those of mothers with primary or secondary education (Figure 3), with a non-significant trend for *z* W/L. There was no difference between children of mothers with primary versus secondary education.

Children from older mothers had lower *z* W/L: −0.31 ± 1.38 (mothers <20 years of age), −0.55 ± 1.20 (mothers 20–29), and −0.89 ± 1.11 (mothers ≥30 years old) (ANOVA, *p* = 0.015). There was an inverse correlation between maternal age and *z* W/L (adjusted *R*^2^: 1.9, *p* = 0.013).

We observed lower anthropometric *z*-scores in families with more children; *z* W/A scores were −0.62 ± 1.19 in one-child families, −0.94 ± 1.30 in families with 2 or 3 children, and −1.14 ± 1.50 in families with 4 or more children (ANOVA, *p* = 0.04). The mean *z* BC/A were: −0.40 ± 0.96, −0.42 ± 1.09, and −0.90 ± 1.29, respectively, for the same groups (ANOVA, *p* = 0.05). There were no significant differences based on place of living or paternal employment.

### 3.4. Feeding Practices

**Figure 4:**
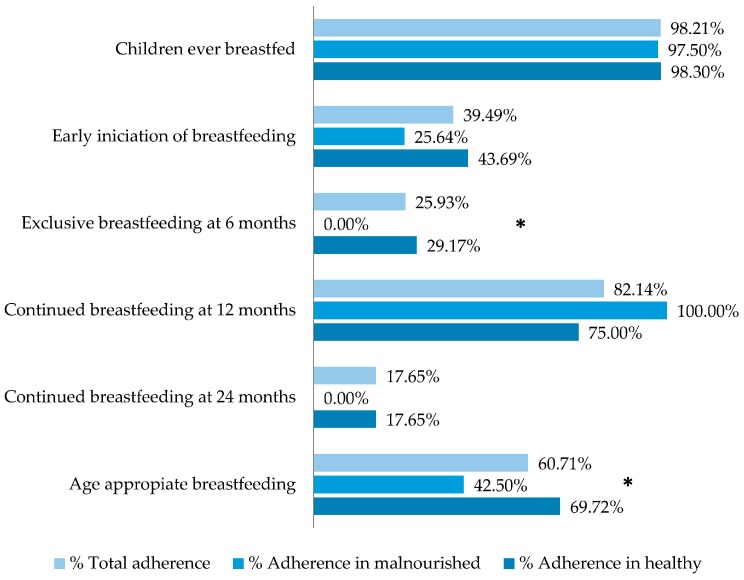
Indicators of breastfeeding practices recommended by WHO in 278 infants seen at the Klinik Saint Espri Health Center, Port au Prince, Haiti (August 2014): Percent adherence in the total group, children with wasting, and healthy children. Footnote: Wasting is defined as W/L <−2 SD according to the WHO 2006 standard. * *p* < 0.05, Chi^2^ test.

**Figure 5:**
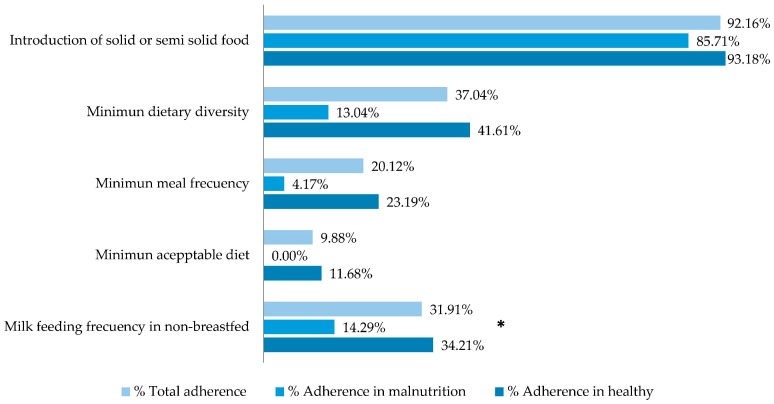
Indicators of adherence to complementary feeding practices (WHO 2008): prevalence in the total group, in children with wasting, and in healthy children. Footnote: Wasting is defined as W/L <−2 SD according to the WHO 2006 standard. * *p* < 0.05 test Chi^2^ test.

The number of cases used to calculate each feeding practice indicator was heterogeneous, given the different sizes of groups by age. Overall adherence was between 98.21% (children ever breastfed) and 3.1% (consumption of iron-rich foods). Adherence to complementary feeding practices was lower than adherence to recommended breastfeeding practices (Figure 4 and Figure 5). Malnourished children were less likely than healthy children to have met recommendations for breastfeeding practices. The differences were significant for: exclusive breastfeeding at 6 months (*p* = 0.032) and age-appropriate breastfeeding (*p* = 0.029). We defined age-appropriate breastfeeding according to the following feeding practice indicators [6]: Infants 0–5 months of age who received only breast milk during the previous day and children 6–23 months of age who received breast milk as well as solid, semi-solid, or soft foods during the previous day, according to the 24-h dietary recall. Adherence to complementary feeding indicators was higher among healthy than malnourished children, although the difference was only significant for “Milk feeding frequency for non-breastfed children”.

The association between adherence indicators and malnutrition is shown in Table 3; more breastfeeding practice indicators than complementary feeding practice indicators were associated with malnutrition. Adherence to many of the recommended feeding practices was significantly lower in children with wasting, stunting, and malnutrition than in healthy children.

## 4. Discussion

The present study shows a high prevalence of malnutrition among Haitian infants and young children seen at an outpatient health center near Port Au Prince. Malnutrition rates were related to several maternal and child characteristics. Associations between malnutrition and adherence to the WHO’s recommended feeding practices varied, with a possible protective effect of breastfeeding. This is the first study exploring this association in a pediatric Haitian population.

This sample of 278 children represented approximately 80% of the children in this age group seen at the Klinik Saint Espri Health Center during the one-month study period. Patients at this health center mainly live in the nearest commune, Croix de Bouquetes [15]. While assessment over a short period may under- or overestimate malnutrition due to seasonal variations in food access, this urban sample is less susceptible to this issue than other populations.

The prevalence of underweight (18.35%) and wasting (13.67%) was higher than in a 2012 national survey [1]: 11.6% and 5.2%, respectively, but stunting was lower: 13.31% vs. 21.90%

Despite changes from the reference standards used in studies since 2010 [16], an observed historical trend [1] reflects a general improvement of nutritional indices in Haiti. Our results are most consistent with data obtained in the most recent studies. The principal differences between our study and others may be attributed to the lower age of our sample: younger than 2 years versus up to 5 years in the national studies cited [1,2,3,5,17]. Previous reports in Haitian children found that that 18.5% were underweight, 10.3% had wasting, and 29.7% had stunting [1,2,5]. Our results were similar except for a higher rate of stunting in the previous study, likely due to the different historical period, the use of the 2006 WHO standard [16], and the inclusion of older children, as stunting is usually a consequence of long-term malnutrition [18,19]. It is also important to consider that children with acute malnutrition are more likely to visit the health services due to more frequent illness or a referral to the Malnutrition Program.

The higher prevalence of wasting in males differs from other international [18] and Latin American reports [20] but is consistent with other from Haiti [3]. Cultural practices that might explain this trend include a higher milk-formula feeding and an earlier introduction to solid foods in male. In addition, higher nutritional impairment in older children is expected [19]. Higher maternal age and larger number of children per family were associated with malnutrition possibly due to progressive poverty in larger families. These risk factors are possibly modifiable by early-life educative interventions and support [21,22]. Maternal education influences the ability to provide adequate food, stimulation and a cleaner environment for the child [23,24]. In the present study, few mothers reported no access to education, a probable underestimation because 15.3% were illiterate and their children had smaller head circumference, indicator of poorer future cognitive function [11,24]. We found association between maternal education and not only of cranial perimeter, but also of H/A, W/A and PB/A, reflecting its importance.

Indicators of adherence to feeding practices in the present study were similar to those reported in a 2005–2006 WHO study [6]; however, that study was conducted nationwide and did not explore the association between malnutrition and feeding practices. There are no data available for comparison at the local level. We found a lower adherence to recommendations regarding continued breastfeeding at two years, age-appropriate breastfeeding, minimum meal frequency, and minimum acceptable diet than the older study. On the other hand, we observed a greater adherence to exclusive breastfeeding until 6 months, predominant breastfeeding until six months, and minimum dietary diversity. An aggressive program to educate and follow up mothers has had a possible positive impact on awareness of the importance of breastfeeding up to six months of age. However, a lack of promotion of continued breastfeeding may have increased the use of breastfeeding substitutes, as shown by the low values for indicators of breastfeeding in infants older than 12 months and age-appropriate breastfeeding. The most specific indicator to assess breastfeeding is exclusive breastfeeding at six months; it shows the greatest in nutritional impact, whereas the other results are mixed. This result is logical, as there is a drop in weight indices at around six months of age, and this indicator groups the children who have managed to continue exclusively breastfeeding until the end of the 0–6-month period. The impact of early initiation of breastfeeding may be reflected in more successful long-term breastfeeding; suggesting an opportunity for an intervention with potential impact at the population level [21,25].

We found a lower prevalence of wasting in children who met feeding recommendations, especially some of the breastfeeding practices. Although some trends were not statistically significant for all complementary feeding practice indicators, it should be noted that improving the diet with currently-available local foods may have a protective effect; such cultural issues must be recognized in designing an intervention [21,22,24,25,26,27,28,29,30,31,32]. However, WHO recommendations [9,10] refer only to food frequency or food groups, not to portions; therefore, real intake may vary according to context, customs, and food availability.

One limitation of our study, with implications for the analysis of the impact of breastfeeding practices, was the difficulty to obtain records for birth weight, gestational age or prematurity. Undernutrition during the first months of life could therefore be overestimated. Since our study was conducted in a limited area within a homogeneous population, we used a convenience sample, prioritizing the recruitment of as many children as possible. Although we compared our results with the reference data available from national surveys of the general population [2,3,4,5], our target population consisted of children seen at a local primary health center [15].

The size of the sample in the present study is lower than other studies in Haiti, but large in relation to the center’s consulting population. These aspects limit partially the application to the general Haitian population.

The reasons for the health center visits were mainly preventive and were not included in this analysis. We did record children who were enrolled in the child malnutrition program, to detect potential biases associated with this variable. This result is not reported in detail, and no additional analyses were performed, as only a small percentage of the sample was enrolled in the program. Exclusion criteria included referral to urgent care, serious illness, and/or clinical dehydration, as these conditions can temporarily lower weight, thereby leading to a false positive diagnosis of malnutrition.

Finally, although the health center is low-complexity, the study population is at greater risk than the general population given the younger age range and the need to visit a health center. This is a relevant analysis at the local level, especially for planning activities designed to prevent and manage malnutrition. The practical implication for the health center is that any child visiting the center is at elevated risk for undernourishment as compared to the national population; and in agreement with other studies [19], the peak of acute malnutrition (and the possibility of timely treatment) occurs before 2 years of age, and more specifically between six and 24 months. By analyzing the association of nutritional status with feeding and lactation practices, we contribute more precise information that can be used locally to develop possible strategies.

As a strength of the study, we developed and administered our own survey after considering studies from other developing countries, selecting items that could better describe this population and contribute to guiding future interventions. The center´s staff participated in and assessed the survey, so the local relevance of the study is strong; however, these findings can also be extrapolated to similar settings. A major difficulty for health centers in developing countries is planning actions beyond everyday contingencies and global recommendations, in a context of limited resources [31,32,33,34]. In the present study, we implemented a simple methodology with low technological and technical support requirements, which allowed us to obtain relevant data from the target population [32].

## 5. Conclusions

In conclusion, we found a significant prevalence of child malnutrition, especially among males and older infants. Maternal illiteracy, older maternal age, and larger numbers of children per family were associated with higher rates of malnutrition. We found that low adherence to WHO-recommended feeding practices was also associated with malnutrition; good breastfeeding practices may be an important protective strategy.

According to evidence-based practices currently recommended by the WHO [21,22,30,33,34,35], our results indicate that the principal recommendations for improving nutritional status in this population are early initiation of breastfeeding, exclusive breastfeeding for infants under six months, continued breastfeeding to two years of age, and complementary feeding beginning at six months and no later. We also recommend strengthening the existing malnutrition program to target the groups of children at the highest risk of malnutrition identified in this study. We hope that our study will contribute to increased awareness in the target population of the Klinik Saint Espri and other health centers and guide child nutrition interventions, using the strengths and resources available in the health center and the community.

## Figures and Tables

**Table 1:** General sociodemographic information in in 278 infants that attended the Klinik Saint Espri Health Center, Port au Prince, Haiti (August to September 2014).

Sociodemographic Characteristics		Percentage
Housing	Located in Croix de Bouquetes	70.77%
	Lightweight material houses or camp	17.9%
Parental work	Any kind of work: formal/informal	34.5%
Parental education	Completed primary education	43.5%
	Illiterate	15.35%
	Never attended to school	6.5%
Number of children	One child	35.7%
	Two or three children	41.8%
	Four or more children	22.5%

**Table 2:** Nutritional status in 278 infants seen at the Klinik Saint Espri Health Center, Port au Prince, Haiti (August to September 2014).

Anthropometric Index	Nutritional Diagnosis	Degree	*z*-Score (WHO 2006)	Prevalence (%)
Weight/Age	Underweight	Severe	*z* W/A ≤ −3	6.12
		Moderate	*z* W/A −2 to −3	12.23
	Normal		*z* W/A −2 to +2	80.22
	Overweight		*z* W/A ≥ +2	1.44
Weight/Length	Wasting	Severe	*z* W/L < −3	3.60
		Moderate	*z* W/L −2 to −3	10.07
	Normal		*z* W/L −2 to +2	84.9
	Overweight and obesity		*z* W/L > +2	1.80
Length/Age	Stunting		*z* L/A < −2	13.31
	Normal		*z* L/A −2 to +2	85.25
	Tall		*z* L/A > +2	1.44
Head circumference/Age	Microcephaly		*z* HC/A < −2	10.80
	Normal		*z* HC/A −2 to +2	86.68
	Macrocephaly		*z* HC/A > +2	2.52
Brachial Circumference/Age	Low		*z* BC/A < −2	4.67
	Normal		*z* BC/A > −2	95.33

**Table 3:** Association between breastfeeding or complementary feeding indicators (WHO, 2010) and malnutrition, in 278 infants and young children seen at the Klinik Saint Espri Health Center, Port au Prince, Haiti (August 2014).

Indicator	Nutritional Status ^#^ (*p*)		
	Wasting	Underweight	Stunting
1. Children ever breastfed	0.92	0.59	0.13
2. Early initiation of breastfeeding	0.21	0.002 *	0.04 *
3. Exclusive breastfeeding under 6 months	0.09	0.32	0.59
4. Predominant breastfeeding under 6 months	0.97	0.000 *	0.02 *
5. Exclusive breastfeeding at 6 months	0.03 *	0.003 *	0.54
6. Continued breastfeeding at 12 months	0.73	0.82	1.00
7. Continued breastfeeding at 24 months	0.16	0.54	nv
8. Age-appropriate breastfeeding	0.03 *	0.20	0.20
9. Introduction of complementary foods	0.83	0.97	0.34
10. Minimum dietary diversity	0.10	0.11	0.90
11. Minimum meal frequency	0.75	0.70	0.90
12. Minimum acceptable diet	0.21	0.049 *	0.35
13. Milk feeding frequency for non-breastfed children	0.02 *	0.06	0.23
14. Consumption of iron rich foods	nv	nv	nv

^#^ Malnutrition was defined according to *z*-scores <−2 SD (WHO 2006 reference). * Chi^2^ test for comparisons against children with normal nutritional status (*z*-scores −2 to +2). *p* < 0.05 was considered significant.

**Figure A1:**
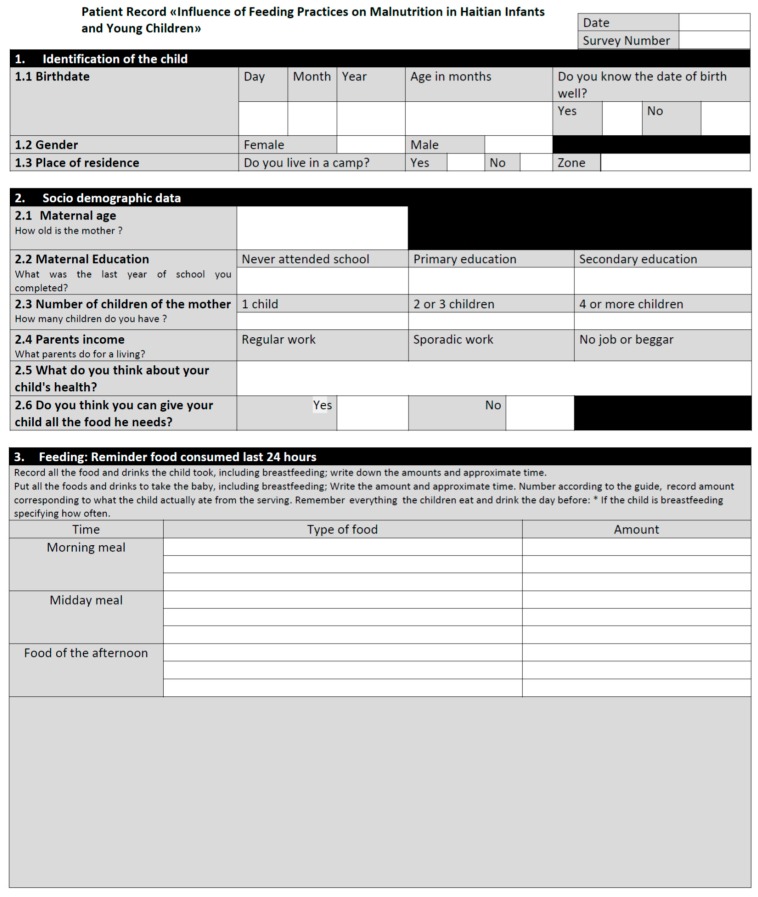

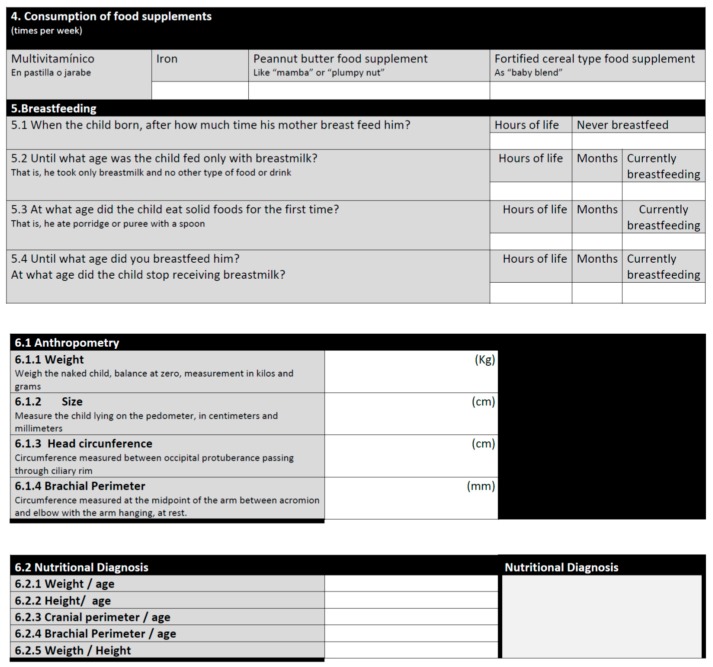
Patient Record <<Influence of Feeding Practices on Malnutrition in Haitian Infants and Young Children>>.

**Figure A2:**
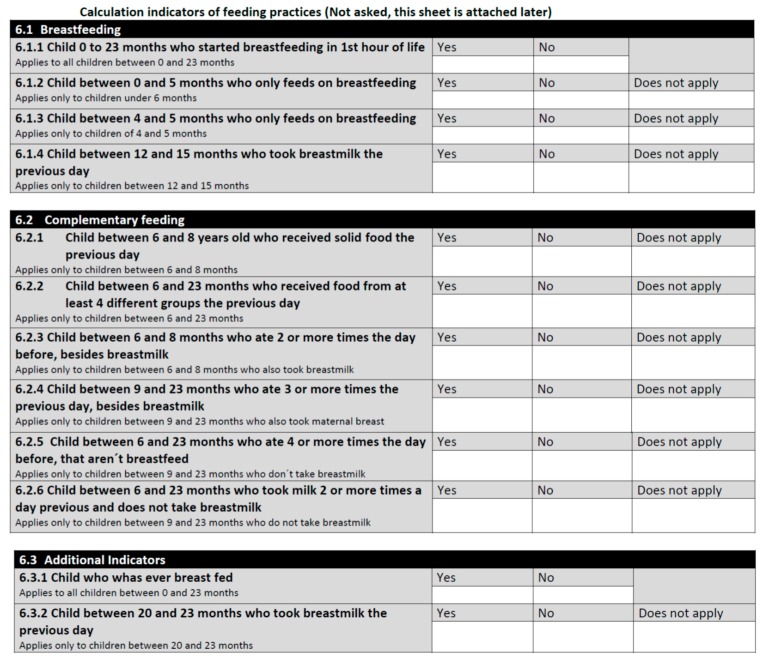
Calculation Indicators of Feeding Practices (Not asked, this sheet is attached later).

**Figure A3:**
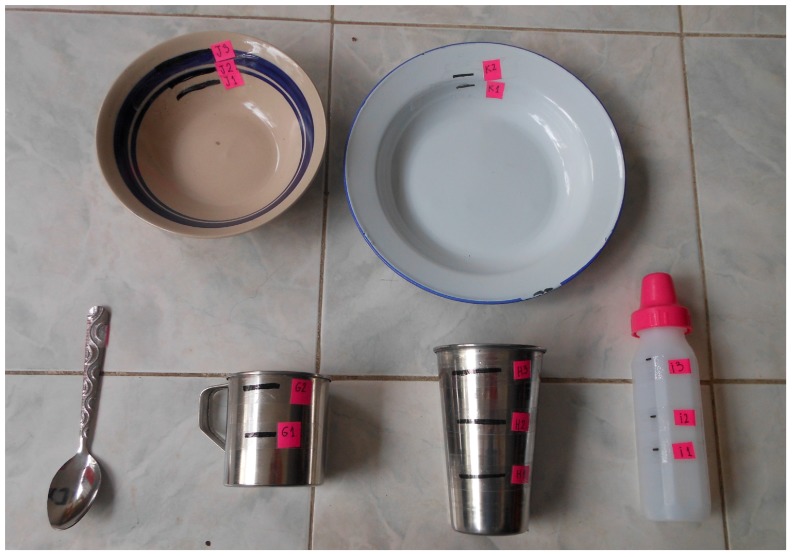
Pictures of plates and glasses from local businesses used for assessing portion size. The plates were code labeled and corresponding volume was mesured.

**Figure A4:**
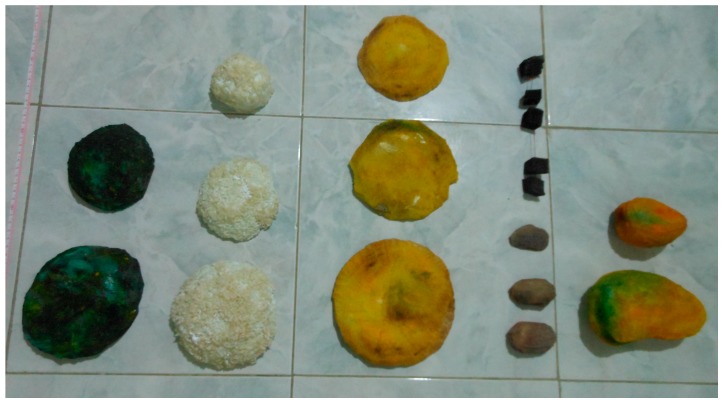
Picture of foam models used to assess portion size, they mimic most common food choices for children in different servings. Models were code labeled.

## Appendix A

Survey used in the study, in Haitian Créole and in English:

1. Identification and general characteristics of the patient
2. Brief social evaluation
3. Nutritional information
  3.1. 24-h dietary recall
  3.2. Use of nutritional supplements
  3.3. Additional breastfeeding questions
4. Anthropometry

Additional page: calculation of individual indicators of feeding practices (WHO 2003).

## Appendix B

Example of common plates and glasses obtained from local businesses and models of common foods with painted plastic foam used for assessing portion size in 24-h dietary recall.

## References

[1] Ministry of health and population Haiti (2012). Rapport de l’enquete Nutritionnelle Nationale Avec la Methodologie SMART. http://mspp.gouv.ht/site/downloads/SMART.pdf.

[2] Ministère de la Santé Publique et de la Population (2007). Enquete Mortalite, Morbidite et Utilisation des Services EMMUS–IV Haiti 2005–2006.

[3] Ministère de la Santé Publique et de la Population (2012). Enquete Mortalite, Morbidite et Utilisation des Services EMMUS–V Haiti 2012.

[4] (2012). World Health Organization (WHO) Country profiles. Haiti Country Profile. http://www.who.int/countries/hti/en/.

[5] Ministry of Public Health and Population. [le Ministère de la Santé Publique and de la Population] (MSPP), Haitian Childhood Institute [l’Institut Haïtien de l’Enfance] (IHE) and ICF International (2013). 2012 Haïti Mortality, Morbidity, and Service Utilization Survey: Key Findings.

[6] World Health Organization (2010). Indicators for Assessing Infant and Young Child Feeding Practices, Country profiles, Part I Definitions. Maternal, Newborn, Child and Adolescent Health.

[7] Dependent on the Church of the Holy Spirit Catholic Church in Memphis, Tenesse. http://www.hspirit.com/.

[8] Sunguya B.F., Poudel K.C., Mlunde L.B., Shakya P., Urassa D.P., Jimba M., Yasuoka J. (2013). Effectiveness of nutrition training of health workers toward improving caregivers’ feeding practices for children aged six months to two years: A systematic review. Nutr. J..

[9] Panamerican Health Organization (2003). Guiding Principles for Complementary Feeding of the Breastfed Child.

[10] World Health Organization (2005). Guiding Principles for Feeding Non-Breastfed Children 6–24 Months of Age.

[11] World Health Organization (2006). WHO Growth Standards for 0–5 Years.

[12] Department of Nutrition, World Health Organization (2011). WHO Anthro: Software for Assessing Growth and Development of the World’s Children.

[13] De Onis M., Yip R., Mei Z. (1997). The development of MUAC-for age reference data recommended by a WHO Expert Committee. Bull. World Health Organ..

[14] De Onis M., Blössner M. (1997). WHO Global Database on Child Growth and Malnutrition.

[15] (2014). Report of Monthly Patient Care Statistics.

[16] WHO Multicentre Growth Reference Study Group (2016). WHO Child Growth Standards: Length/Height-for-Age, Weight-for-Age, Weight-for-Length, Weight-for-Height and Body Mass Index-for-Age: Methods and Development.

[17] Black R.E., Allen L.H., Bhutta Z.A., Caulfield L.E., de Onis M., Ezzati M., Mathers C., Rivera J. (2004). Maternal and Child Undernutrition Study Group. Maternal and child undernutrition: Global and regional exposures and health consequences. Lancet.

[18] Lozano R., Naghavi M., Foreman K., Lim S., Shibuya K., Aboyans V., Abraham J., Adair T., Aggarwal R., Ahn S.Y. (2012). Global and regional mortality from 235 causes of death for 20 age groups in 1990 and 2010: A systematic analysis for the Global Burden of Disease Study 2010. Lancet.

[19] Victora C.G., de Onis M., Hallal P.C., Blössner M., Shrimpton R. (2010). Worldwide timing of growth faltering: Revisiting implications for interventions. Pediatrics.

[20] Kac G., Alvear J.L.G. (2010). Malnutrition in Latin America Network Program of Science and Technology for Development. Epidemiology of malnutrition in Latin America: Current situation. Nutr. Hosp..

[21] Haroon S., Das J.K., Salam R.A., Imdad A., Bhutta Z.A. (2013). Breastfeeding promotion interventions and breastfeeding practices: A systematic review. BMC Public Health.

[22] Dewey K.G., Adu-Afarwuah S. (2008). Systematic review of the efficacy and effectiveness of complementary feeding interventions in developing countries. Matern. Child Nutr..

[23] Prado E.L., Dewey K.G. (2014). Nutrition and brain development in early life. Nutr. Rev..

[24] Imdad A., Yakoob M.Y., Bhutta Z.A. (2011). Impact of maternal education about complementary feeding and provision of complementary foods on child growth in developing countries. BMC Public Health.

[25] Renfrew M.J., McCormick F.M., Wade A., Quinn B., Dowswell T. (2012). Support for healthy breastfeeding mothers with healthy term babies. Cochrane Database Syst. Rev..

[26] Heckert J., Boatemaa S., Altman C.E. (2014). Migrant youth’s emerging dietary patterns in Haiti: The role of peer social engagement. Public Health Nutr..

[27] Diop El H.I., Dossou N.I., Ndour N.M., Briend A., Wade S. (2003). Comparison of the efficacy of a solid ready-to-use food and a liquid, milk-based diet for the rehabilitation of severely malnourished children: A randomized trial. Am. J. Clin. Nutr..

[28] Ciliberto M.A., Sandige H., Ndekha M.J., Ashorn P., Briend A., Ciliberto H.M., Manary M.J. (2005). Comparison of home-based therapy with ready-to-use therapeutic food with standard therapy in the treatment of malnourished Malawian children: A controlled, clinical effectiveness trial. Am. J. Clin. Nutr..

[29] Bisits Bullen B.A. (2011). The positive deviance/hearth approach to reducing child malnutrition: Systematic review. Trop. Med. Int. Health.

[30] Picot J., Hartwell D., Harris P., Mendes D., Clegg A.J., Takeda A. (2012). The effectiveness of interventions to treat severe acute malnutrition in young children: A systematic review. Health Technol. Assess..

[31] Uauy R. (2008). Undernutrition is undernourished. Public Health Nutr..

[32] Pridmore P., Carr-Hill R. (2011). Tackling the drivers of child undernutrition in developing countries: What works and how should interventions be designed?. Public Health Nutr..

[33] World Health Organization and UNICEF (2003). Global Strategy for Infant and Young Child Feeding.

[34] World Health Organization (2013). Essential Nutrition Actions: Improving Maternal, Newborn, Infant and Young Child Health and Nutrition.

[35] Ward K.N., Byrne J.P. (2011). A critical review of the impact of continuing breastfeeding education provided to nurses and midwives. J. Hum. Lact..

